# The Dangers of Celebrity

**Published:** 1888-04-21

**Authors:** 


### THE DANGER OF CELEBRITY.

We have been requested to publish the following: "The celebrated chemist, Justus von Liebig, it is well known, made a number of most important discoveries, but, well foreseeing the danger that might arise by applying his name wrongfully to a number of chemical articles invented by himself, he confined the right of using his name exclusively to the well-known Liebig's Extract of Meat Company (Limited), upon the distinct understanding that himself and his delegate, Prof. Max von Pettenkofer, should analyse and control the whole of the Company's production of Extract of Meat. When English Courts of Law decided that anybody might use Liebig's name for any Extract of Meat purporting to have been manufactured according to his process, and of whatever quality it might be, Baron Liebig's sense of justice and fairness was naturally shocked and surprised, for this decision enabled persons to sell, under the title of Liebig's Extract of Meat, an article which was not manufactured according to his special directions, or under the control of himself or his delegate. In France and Belgium the Courts held otherwise, and if a purchaser buys Liebig's Extract of Meat in those countries, he obtains that manufactured by the Company. Extracts of Meat of most inferior qualities are sold under the name of 'Liebig's.' A number of other articles, such as 'Liebig's beef wine,' 'Liebig's perfect health sweet,' 'Liebig's perfect health lozenges,' 'Liebig's extract of malt,' 'Liebig's infant food,' &c., are all articles with which Baron Liebig has not the slightest connection. The public, in its own interest, cannot, therefore, be sufficiently warned against purchasing any of those articles, in the belief that they have been invented or guaranteed by the celebrated and world-renowned chemist, Baron Justus von Liebig."

